# Open controversies on the treatment of undescended testis: An update

**DOI:** 10.3389/fped.2022.874995

**Published:** 2022-07-27

**Authors:** Jie Liu, Wenli Xiu, Bangzhi Sui, Zhiyuan Jin, Xudong Xu, Nan Xia, Guangqi Duan

**Affiliations:** ^1^Department of Pediatric Surgery, Yijishan Hospital of Wannan Medical College, Wannan Medical College, Wuhu, China; ^2^Institute of Digital Medicine and Computer-Assisted Surgery of Qingdao University, Qingdao University, Qingdao, China; ^3^Department of Pediatric Surgery, The Affiliated Hospital of Qingdao University, Qingdao University, Qingdao, China; ^4^Shandong Provincial Key Laboratory of Digital Medicine and Computer-Assisted Surgery, Qingdao, China

**Keywords:** cryptorchidism, children, diagnosis, inguinal, surgery

## Abstract

Cryptorchidism is a common congenital malformation in pediatric urology. Although there have been many studies on the etiology of the disease, it has not been fully clarified, and while its diagnostic and treatment models have gradually approached standardization and systematization, some controversies regarding treatment remain. Additionally, although ultrasound is a non-invasive examination without ionizing radiation, its role in the evaluation of cryptorchidism remains controversial. The main basis for treating cryptorchidism is orchidopexy, and the main view on treatment age is that treatment should be performed between 6 and 12 months after birth, but no more than 18 months after birth. The view on hormone therapy is still controversial because most scholars believe that early surgery is the key to treatment. There are many surgical treatment methods for cryptorchidism, including traditional open surgery and laparoscopic surgery, which provide satisfactory results. In conclusion, the treatment of undescended testis (UDT) had been largely standardized, apart from the treatment of high intra-abdominal testis (IAT), which remains a matter of debate.

## Introduction

Cryptorchidism is a common congenital malformation in pediatric urology which is also known as undescended testis (UDT) or incomplete testicular descent (1–3). According to the location of the testis, cryptorchidism can be divided into palpable testis and non-palpable testis (NPT), and palpable testis accounts for more than 80% of cryptorchidism cases (4). Cryptorchidism is associated with testicular tumors and hernia, and thus, it requires active treatment (5).

Although there have been many studies on the etiology of the cryptorchidism, it has not been fully clarified, and while its diagnostic and treatment models have gradually approached standardization and systematization, some controversies regarding treatment remain, including (i) the effectiveness, role, and status of hormone therapy in the treatment of cryptorchidism; (ii) when the intervention should be performed and whether early surgery can reduce the risk of testicular carcinogenesis; and (iii) whether retractable testis (RT) requires systematic clinical observation and intervention. In addition, there is still great controversy regarding the best surgical treatment for palpable testis, especially for high intra-abdominal testis (IAT). In the recent years, with the progress of basic and clinical research, scholars have made achieved a greater understanding of the disease. This review summarizes the recent progress that has been made in the diagnosis and treatment of cryptorchidism.

## Etiology of cryptorchidism

Testicular descent occurs between 8–15 and 25–35 weeks of gestation, during two hormone-controlled stages (6). In the fetal period, both testes may not descend to the scrotum on time for various reasons, which can manifest as cryptorchidism at birth. In boys with cryptorchidism, there is still the possibility of spontaneous testicular descent within 6 months after birth (7). The incidence rate of cryptorchidism has gradually increased in the recent years (8). Cryptorchidism may be closely related to genetics, hormone synthesis and secretion, premature delivery, pregnancy in older women, and the environment (9). According to the statistics, the incidence rate of cryptorchidism in preterm infants is 18–30%, and 0.8–20% of full-term neonates can also have symptoms of cryptorchidism; cases of unilateral cryptorchidism are common (7, 8).

Wang et al. (10) analyzed the clinical and genetic characteristics of 64 cases of congenital hypogonadotropic hypogonadism (CHH) and found solitary cryptorchidism in 7% of the cases and cryptorchidism with small penis in 39% of the cases. The results showed that the inactivation of CHH-related genes may be related to cryptorchidism. Chai et al. (11) examined both the phenotypical evolution and comparative genomics of 380 cryptorchidism-related genes in mammals and found not only that scrotal testis is the ancestral state of mammals but also that ascrotal testis has evolved independently many times. In addition, the rapidly evolving and actively expressed genes found by the researchers suggest that the derived status of the ascrotal incompletely descended testis (IDT) and UDT phenotypes in mammals can be attributed to the adaptive evolution of genes involved in testicular descent and muscle development.

In a study of mice by Ardiani et al. (5), reactive oxygen species were found to be involved in the occurrence of cryptorchidism. Supplementation with erythropoietin improved cryptorchidism-induced damage to germinal epithelial cells and spermatogenesis. Endocrine disruptors (EDs) are exogenous substances that can damage the endocrine system. Cargnelutti et al. (12) discussed the adverse effects of exposure to EDs on fetal testicular development, male puberty, and transition age. Although the current evidence did not clarify the impact of EDs on human male reproductive health and the results were controversial, the general conclusion pointed to a positive correlation between exposure to EDs and reproductive system damage. Desalegn et al. (3) performed a prospective cohort study in Norway that recruited 2,606 pairs of mothers and infants and ultimately included 1,326 pairs of mothers and children. Measurements of 27 potential EDs in breast milk were investigated as the alternative indicators of perinatal exposure and cryptorchidism risk. The results showed that perinatal exposure to polychlorinated biphenyls (PCB)-74, PCB-114, PCB-194, and β-hexachlorocyclohexane (β-HCH) was associated with an increased risk of congenital cryptorchidism. Estors et al. (13) conducted a case–control study of 103 boys with cryptorchidism and 107 boys with hypospadias. The results showed that old age, parental occupational exposure to EDs, drug abuse, smoking, and paternal history of urinary disease may increase the risk and predict the development of these malformations. However, the authors also noted that a large-sample study may be needed to analyze the relationship between occupational exposure to individual EDs and drugs and these urinary malformations. The incidence rate of cryptorchidism in boys was assessed by Mitsui et al. (14) in Japan; a total of 51, 316 newborn males were recruited, and the incidence rate of cryptorchidism in male newborns was analyzed based on 14 parental occupations. The results showed that maternal age and smoking during pregnancy affected the incidence rate of cryptorchidism, whereas the working environment of parents had no significant effect on the incidence rate of cryptorchidism. However, scholars also believe that the study may underestimate mild and transient cryptorchidism. Additionally, it is still necessary to further study the risk factors for cryptorchidism related to parental occupation. However, Gurney et al. (6) studied a series of hypothetical risk factors that have been identified as potentially related to the development of cryptorchidism. Only a few factors showed consistent evidence of being related to cryptorchidism. Factors with clear evidence might be the components of the real causal exposure. The study further found that the relative importance of each risk factor may vary greatly between mother and child pairs and that all these factors may vary from region to region.

## Diagnosis of cryptorchidism

### Endocrine diagnosis of cryptorchidism

Grinspon et al. (15) studied the anti-Müllerian hormone (AMH) level and testicular function in prepubertal cryptorchid boys. A total of 1, 557 patients in the database met the search criteria, and 124 patients with unilateral cryptorchidism and 186 patients with bilateral cryptorchidism were randomly selected. The results showed that AMH production was decreased in boys with prepubertal cryptorchidism, especially boys with bilateral cryptorchidism. Although the serum AMH level may be in the normal range, the incidence of testicular dysfunction in childhood is also quite high under this common condition. In the prospective cohort study conducted by Koskenniemi et al. (16), 2,545 boys were recruited prenatally, their testicular position was examined, and the testicular distance to pubic bone (TDP) was recorded. Finally, 680 Danish boys and 362 Finnish boys were included in the study. The researchers also measured the serum concentration of reproductive hormones and insulin-like growth factor-I (IGF-I) at 3 months of age. The results showed that the TDP increased from birth to 3 months of age and then decreased. Length, gestational age, gestational age weight, and penis length were positively correlated with TDP. The researchers also found that the IGF-I concentration, inhibin B/follicle-stimulating hormone ratio, and testosterone/luteinizing hormone ratio were independently and positively correlated with the TDP. Furthermore, Lee et al. (17) reported that it is very useful to include the usefulness of AMH determination in patients with non-palpable gonads, to make a differential diagnosis between intraabdominal testes and anorchia.

### Physical examination diagnosis of cryptorchidism

At present, the diagnosis of cryptorchidism in children is mainly based on physical examination. Physical examination can be used to not only diagnose cryptorchidism and distinguish palpable testis from NPT but also identify RT (18). During the examination, each testis should be pushed through the internal inguinal ring to the scrotum and examined carefully; the posture of the examinee can be standing or supine. It is recommended to that this check is performed repeatedly in multiple locations. To avoid false positives caused by the testicular reflex of boys in response to a low examiner hand temperature, the hands of examiners should be warmed before the examination.

### Imaging examination diagnosis of cryptorchidism

Although ultrasound is a non-invasive examination without ionizing radiation, its role in the evaluation of cryptorchidism remains controversial. The guidelines of the Canadian Urological Association-Pediatric Urologists of Canada (CUA-PUC) published by Braga et al. (19) posit that an imaging examination for cryptorchidism is not cost effective and may delay referral and surgical treatment; thus, it cannot be recommended as a standard auxiliary means for the pre-operative evaluation of these children. However, some scholars have different views. Tasian et al. (20) studied the practicality, indications, and effectiveness of diagnostic imaging for cryptorchidism and found that ultrasound is the most commonly used imaging method for evaluating cryptorchidism, but the ability of ultrasound to reach the testis varies, with a sensitivity and specificity of 45 and 78%, respectively, for accurate NPT localization. The researchers believe that although diagnostic imaging plays a limited role in the routine evaluation of most children with cryptorchidism, ultrasonography can help to identify the possible Mullerian structures in children with sexual development disorders or hypospadias complicated with cryptorchidism. In 2020, You et al. (21) reported a study on the clinical value of measuring ultrasonic activity and the testicular atrophy index (TAI) by three-dimensional ultrasound imaging in the pre-operative and post-operative evaluation of cryptorchidism. The results showed that the TAI can objectively reflect the recovery of testicular volume after cryptorchidism surgery, and the evaluation of ultrasonic activity is helpful for the clinical treatment of the disease. Shibata et al. (22) conducted a study on the accuracy of contralateral testicular hypertrophy in predicting absent testis in Japanese boys with NPT. The data showed that contralateral testicular hypertrophy strongly indicated absent testis in Japanese boys. The optimal cutoff for contralateral testicular hypertrophy with calipers was 21 mm and 1.6 cm^3^. They believe that this result provides valuable information for pre-operative consultation and treatment planning. Berger et al. (23) reported that for NPT, inguinal ultrasound and measurement of the contralateral testis have a synergistic effect in predicting the surgical approach. Adding an ultrasound examination to the clinical examination can significantly improve the prediction of the correct surgical method and avoid an unnecessary laparoscopic examination. Wei et al. (24) conducted a retrospective study on the accuracy of contralateral testicular hypertrophy in Chinese boys of different ages in predicting the fate of NPT for 18 years at a single center. The results showed that testicular volume > 0.65 ml or testicular length > 16.55 mm measured by ultrasound could predict monorchidism with an accuracy of approximately 65%. In young patients aged 0–2 and 4–6 years, the overall predictive accuracy increased to approximately 73%, but the authors pointed out that laparoscopic exploration is still needed. In addition, Mah et al. (25) conducted an electronic survey of 339 pediatric urologists to evaluate the decisions made for the management of NPT and determine the impact of contralateral testicular size, sonographic findings, surgeon region, and years in practice. The results showed that contralateral testicular hypertrophy and ultrasonography had no significant effect on the application of inguinal/scrotal exploration.

Although computed tomography (CT) has been used in some studies of cryptorchidism, it is particularly rare in pediatric population. In addition to the defects of ionizing radiation, the main reason is the need for anesthesia, which limits the application of CT in the diagnosis of cryptorchidism in boys (26). Although there is no ionizing radiation in magnetic resonance imaging (MRI), however, MRI is expensive, and it is difficult for children to cooperate in the examination process; in some cases, sedation or anesthesia is also required. Fazal et al. (27) conducted a cross-sectional study of 416 patients diagnosed with NPT at the time of physical examination at a tertiary hospital in Pakistan. All boys underwent abdominal, pelvic, and scrotal ultrasonography before laparoscopic exploration, as well as pre-operative abdominal and pelvic diffusion-weighted (DW)-MRI using a 1.5-T MRI system and body coil. Taking laparoscopy as the gold standard, if an atrophic testis was diagnosed in the abdominal cavity, laparoscopic orchiectomy was performed, and the orchiectomy specimens were subjected to histopathological examination. The results show that DW-MRI can improve the detection rate of cryptorchidism. The researchers reported that DW-MRI can be used as a better recommended imaging tool in terms of pre-operative diagnostic accuracy in NPT localization. However, Carpenter et al. (28) reported that an imaging examination before a referral is unnecessary and that more efforts need to be made to train primary healthcare providers and promote more cost-effective and timely care for cryptorchid boys.

In conclusion, ultrasound, CT and MRI are only auxiliary examinations with limited roles in cryptorchidism in boys and may not be used to apply selection criteria for diagnosis and surgical planning.

## Treatment of cryptorchidism

### Hormone therapy

The theoretical basis of hormone treatment for cryptorchidism is that hypothalamic–pituitary–gonadal (HPG) axis hormone deficiency can lead to cryptorchidism, whereas human chorionic gonadotropin (hCG) and gonadotropin-releasing hormone (GnRH) can stimulate the synthesis of testosterone and promote the testicular descent to the scrotum (29, 30). Many studies have shown that hCG therapy and luteinizing hormone-releasing hormone (LHRH) or GnRH therapy are more effective in the hormone treatment of cryptorchidism (31, 32). The effect of hCG on testicular stromal cells can promote testosterone synthesis. The World Health Organization (WHO) proposed that the effective dose of hCG in the treatment of cryptorchidism is 500 U per intramuscular injection for patients 1–6 years old and 1,000 U per intramuscular injection for patients over 6 years old, two times a week for 5 weeks (33). Li et al. (34) pointed out in a meta-analysis that LHRH is effective in the treatment of cryptorchidism, especially in cases of inguinal or prescrotal testis. The testicular descent rates in the LHRH treatment group and placebo group were 20.9 and 5.6%, respectively (1.2 mg LHRH daily for 4 weeks). Vincel et al. (35) conducted a prospective study of 10 boys with isolated bilateral cryptorchidism and defective mini-puberty to evaluate the efficacy of GnRH in restoring defective mini-puberty. The boys were randomly divided into two groups. Those in the control group underwent a second orchidopexy without hormone therapy. Those in the GnRH group underwent GnRH treatment and then a second orchidopexy. The results showed that GnRH treatment was effective in restoring the defective puberty of boys with bilateral cryptorchidism. Elsherbeny et al. (36) analyzed 75 boys with cryptorchidism who were treated with hCG. The results showed a low success rate of hCG in the treatment of cryptorchidism, which was attributed to the anatomical abnormality related to cryptorchidism.

Some scholars believe that in addition to the inefficiency of hormone therapy, there is a lack of long-term data, the quality of relevant research is usually low, and different treatment schemes have been investigated in different research populations. These views are seriously biased. In fact, the efficacy of surgery may not be better, and the quality of surgical studies results is as low as that of hormone studies (37–39). The Nordic consensus issued in 2007, the American Urological Association guidelines issued in 2014, and the European urological guidelines issued in 2016 do not recommend the use of hormone therapy to induce testicular descent in boys with cryptorchidism. Orchidopexy is recommended as the preferred treatment (37–39). However, all there expert guidelines are not evidence-based (i.e., have not used the GRADE system for recommendations). Therefore, there is a high risk of bias in these guidelines.

In addition, there are meta-analyses and other reviews indicating that hormonal treatment may be efficacious when used in cryptorchidism with testes in mid to low inguinal position. Pyörälä et al. analyzed 33 articles published in English between 1958 and 1990 to evaluate the role of LHRH and hCG in the treatment of cryptorchidism. A total of 3,282 boys and 4,524 cryptorchidism were included in the analysis. The results showed that LHRH was effective in the treatment of cryptorchidism. hCG was more effective than placebo, but there were few data on its efficacy (40). A systematic review conducted by Henna et al. (41) on the treatment of hormonal cryptorchidism: a meta-analysis of randomized clinical trials showed evidence that LHRH was more effective than placebo. However, because this evidence is based on a few trials, the sample size is small, and the risk of bias is moderate, it is impossible to recommend this treatment to all people, and there is no evidence to support the use of hCG in a larger dose and interval. Hadziselimovic et al. believe that hormone therapy is still the first choice for the treatment of cryptorchidism. It eliminates the need for subsequent surgery. In case of no response, it is conducive to testicular fixation and greatly helps to reduce the incidence of complete testicular atrophy after unilateral and more serious bilateral surgery. The authors highly recommend hormone therapy for cryptorchidism boys in the high infertility and azoospermia risk group who successfully underwent testicular fixation at an early stage (42). In conclusion, there is still controversy about hormone therapy for cryptorchidism.

### Surgical treatment

#### Age at surgical intervention

At present, the mainstream opinion regarding the age of treatment for cryptorchidism is that if the testis does not descend to the scrotum by 6 months after birth, it is recommended that treatment is performed as soon as possible, preferably before the age of 12 months and no later than the age of 18 months (43–45). One study reported that the rate of testicular atrophy did not increase after early surgical intervention (46). In addition, research has shown that delaying treatment may affect fertility-related spermatogenesis and hormone secretion in adulthood and potentially the occurrence of tumors (47, 48). Kollin et al. (49) conducted a randomized controlled study of 72 boys with cryptorchidism who underwent surgical treatment at the age of 9 months and 83 boys who underwent surgery at the age of 3 years. The testicular volume was monitored on follow-up with B-mode ultrasound after the operation. The results showed that the testicular volume can be used as an index to indirectly evaluate the likelihood of future spermatogenesis. Additionally, compared with treatment at the age of 3, surgical intervention at the age of 9 months showed significant benefits for testicular growth. However, there are serious studies by Hadziselimovic et al. (50) showing that it is not the age at surgery but the functional state of the testes is predictive of future testicular capacity. This study provides evidence of higher quality than that of Kollin et al. (49) cited by the authors. Indeed, while Kollin et al. (49) did a prospective randomized study, they only analyzed the testes during childhood, whereas Hadziselimovic et al. (50) analyzed the patients after a longitudinal follow-up to adulthood. Tseng et al. (51) analyzed the testicular volume of 134 boys with unilateral cryptorchidism in different age groups using the growth percentage ratio (GPR) of the affected and normal testes. The average follow-up time of the study was longer than 3.9 years. The results showed that compared with other age groups, faster testicular growth occurred after orchidopexy before 1 year of age. The authors suggest that according to the relevant clinical data and the rate of testicular growth, surgery should be performed in children with cryptorchidism before the age of 1 year. Xu et al. (52) analyzed the pathological reports of testicular specimens of boys with cryptorchidism aged 10 or above at a pediatric hospital from 1994 to 2016. The results showed malignant changes in 12.5% of boys with IAT but not in boys with extra-abdominal testis. The authors reported that orchiectomy or biopsy was necessary for older children with IAT but extra-abdominal testis required different treatment.

Wei et al. (53) analyzed the operation age of 3, 784 children undergoing orchidopexy from 1993 to 2014. The median age was 4 years old from 1993 to 2000 and decreased to 3 years old from 2000 to 2010. Furthermore, although the median age showed a downward trend from 2010 to 2014, it was still >18 months. In 2020, Jay et al. (54) collected the data of all male infants diagnosed with cryptorchidism born in England, Finland, Scotland, Canada, Sweden, Australia, and Iceland from 2003 to 2011, investigated the compliance of these regions with the 2008 guidelines, and studied the potential socioeconomic inequality in the timing of surgery between 1 and 3 years of age. The results showed that before the introduction of the guidelines (44, 45), there was limited evidence of socioeconomic inequality in the surgical treatment of cryptorchidism. Among boys born after 2008, the proportion of boys who underwent orchidopexy in the highest and lowest socioeconomic groups in different jurisdictions varied, with an absolute difference of 2.5–5.9%, indicating that the application of orchidopexy was unfair, with a tendency to be performed for patients of a higher socioeconomic status before the age of 1 year. The study found a consistent lack of compliance with the guidelines in various jurisdictions that raised questions about the appropriateness of the guidelines.

#### Palpable testis treatment

In orchidopexy for palpable testis, open or laparoscopic surgery can be used. Open surgery can be performed using two approaches: an inguinal incision and a single incision through the scrotum (Bianchi technique) (55). Ali et al. (56) performed the surgery with the single-incision Bianchi technique on 100 boys with cryptorchidism aged 6–12 months. The testicles of all boys were successfully transferred down to the scrotum, and the operation time was 20–36 min. There were no cases of testicular atrophy or ascent. The results showed that compared with the inguinal approach, the unilateral Bianchi technique shortened the operation and yielded similar success and complication rates with better aesthetics. The Bianchi technique is sufficient for treating palpable testicles, especially in 6-month-old infants. McGrath et al. (57) conducted a randomized controlled study to compare the effects of scrotal and inguinal orchidopexy on the analgesic demand, post-operative pain score, and complication rate. The results showed that although the average post-operative pain score and analgesic consumption of patients treated with scrotal orchidopexy were slightly lower, the degree of pain on all scales was mild. Additionally, the median analgesic consumption at home and the pain score, operation time, and complication rate were similar in the two groups. The authors considered that scrotal orchidopexy is an effective alternative to inguinal orchidopexy in cases of low cryptorchidism. Wang et al. (58) reported the successful treatment of 796 boys with middle and low cryptorchidism by orchidopexy with a transverse scrotal incision. The authors considered the transverse scrotal incision to have the advantages of causing less trauma and yielding a more aesthetic post-operative scar and to thus be a safe and feasible method for the treatment of middle and low cryptorchidism. Yu et al. (59) published a systematic review and meta-analysis comparing the Bianchi technique with traditional two-incision inguinal orchiopexy in the treatment of palpable testis in children in 2022. The results showed that the Bianchi technique was a safe, effective, promising, and potentially minimally invasive method for the treatment of palpable testis in children. Compared with traditional two-incision inguinal orchiopexy, the Bianchi technique has the advantages of a shorter operation time, a shorter hospital stay, less post-operative pain, and a good cosmetic effect, with no increase in the incidence of short- or long-term complications. For some patients with cryptorchidism, especially those with low cryptorchidism, the Bianchi technique is considered an alternative to traditional two-incision inguinal orchiopexy.

With the development of laparoscopic technology, scholars have begun to use laparoscopy to treat children with palpable testis, with satisfactory results. Riquelme et al. (60) reported the treatment of 28 patients with testicular cryptorchidism by laparoscopic surgery in 2006. The results showed that this technology was not associated with more complications than open surgery. Additionally, Riquelme et al. (61) reported 15 years of experience in the laparoscopic treatment of 155 patients with palpable testis. The patients were followed up for 6 months to 15 years, and no cases of testicular atrophy were found. The authors reported that laparoscopy is a very safe option for patients with palpable testis, regardless of the position in the inguinal canal. You et al. (62) reported the treatment of 773 cases by laparoscopy at a tertiary medical institution in China and highlighted the obvious advantages of the minimally invasive nature of laparoscopy. In addition, cases of a contralateral patent processus vaginalis (PPV) can be identified and treated at the same time to avoid the occurrence of metachronous inguinal hernia. Escarcega-Fujigaki et al. (63) conducted a prospective, comparative, observational, longitudinal, and double-blinded study in Mexico in which 63 patients with testicular cryptorchidism underwent surgery. The results showed that both laparoscopic and open surgery yielded satisfactory results, with no significant differences. Thus, they concluded that the technique can be chosen by the surgeon based on the surgeon’s laparoscopic experience and training. Gu et al. (64) conducted a retrospective study of 291 patients with palpable testis. A total of 170 patients underwent laparoscopic treatment, and 121 patients underwent traditional open surgery. Review of the patient age, operation time, and clinical results suggested that laparoscopic treatment is an appropriate choice for cryptorchidism with palpable testis, especially in children under 2 years old. However, the success rate of laparoscopic treatment decreased with age. A multi-institutional prospective randomized study on the treatment of peeping testis conducted by Elderwy et al. (65) showed fairly comparable results with open and laparoscopic testicular fixation. However, laparoscopy was more effective because two repeat orchiopexies were required in the open surgery group. Anand et al. (66) conducted a systematic review and meta-analysis on the efficacy of laparoscopic testicular fixation in the treatment of palpable testis in 2021. The final meta-analysis included five studies involving 705 children, with 369 in the laparoscopic group and 336 in the open group. The results showed no significant difference in the operation time or rate of repeat testicular fixation, early complications, or testicular atrophy between the two groups. However, in most studies, laparoscopy was associated with higher costs. The authors concluded that the laparoscopic technique is safe and effective for children with palpable testis.

In short, with the development of minimally invasive technology, a variety of surgical methods have become available for the treatment of palpable testis. The appropriate surgical method can be selected according to the operator’s surgical experience, the equipment at medical institutions, and the specific situation of patients.

In RT, the testis is generally considered to be located in the upper scrotum or lower inguinal canal but can be completely lowered into the scrotum through manual reduction without resistance and restored to the original position through the testicular levator reflex (67). Cryptorchidism should be distinguished from RT. Scholars have different views on the diagnosis and treatment of RT. Goede et al. (68) used ultrasound to measure the testicular volume of 157 boys aged 0.8–11.5 years, including 92 normal boys and 65 boys diagnosed with RT. The measured volume values were compared, and the results showed that the volume in boys with RT was significantly less than the recently determined standard value. Anderson et al. (69) conducted a prospective study of 20 boys with RT (1–12 years old) and 25 human fetuses with the testes in the scrotal position. The age range of the fetuses was 26–35 weeks of gestation. The results showed that the risk of PPV and epididymal abnormalities (EAs) in patients with RT was significantly higher than that in normal fetuses. Hori et al. (70) retrospectively analyzed the cases of 215 boys diagnosed with RT; among them, 89 showed spontaneous improvement, 43 underwent orchidopexy, and 13 remained under follow-up. The results showed that the follow-up time from diagnosis to remission was significantly longer in the spontaneous remission group than in the surgical intervention group and that there were significantly more cases of spontaneous improvement of bilateral RT than of unilateral RT. Spontaneous improvement was observed in boys of all ages by was significantly more common in patients younger than 3 years. The authors considered RT not to be a variant of normal testicles. The early examination of children is very important to prevent a missed diagnosis, misdiagnosis, and unnecessary surgery. To further study the diagnosis of RT, the same scholar published a retrospective study on useful objective variables and factors for the diagnosis of RT and cryptorchidism in 2022 (71). The results showed that the age of the patient and laterality and location of the testis at the time of examination may be helpful for the diagnosis of RT and cryptorchidism. At present, for RT, the main recommendation is to regularly monitor the size and shape of the testicles until puberty. If the testicles are located in the scrotum without retraction during the observation process, treatment is not needed.

#### Non-palpable testis treatment

In the recent years, great progress has been made in the treatment of NPT. The surgical methods include traditional open surgery and laparoscopy-assisted orchidopexy. Arena et al. (72) reported the treatment of 21 cases of inguinal testicular fixation without spermatic cord vascular division in the treatment of IAT. All patients underwent ultrasonography before the operation. When no testis was found by ultrasonography, laparoscopic exploration was performed. If the testis was found under laparoscopy, the operation was resumed through an inguinal incision. All boys underwent inguinal orchidopexy. At the 1-week follow-up, four testes were in the high scrotal position. At the 6-month follow-up, one testicle was in the high scrotal position, and another testicle had retracted to the outer inguinal ring. There were no cases of testicular atrophy. It is considered that inguinal orchidopexy is a safe, reliable, and successful surgical method for the treatment of IAT that should take precedence over techniques that require vascular division because of the high incidence of atrophy. Chan et al. (73) retrospectively studied the experience in treating NPT with laparoscopy as the initial surgical method for 10 years; 339 boys with NPT underwent laparoscopy as the first examination. The results showed that laparoscopic surgery for boys with NPT is a safe method that can also guide the follow-up groin exploration. In boys with unilateral NPT, laparoscopy can improve the probability of success of groin exploration, whereas orchidopexy can be successfully performed in 90% of children with bilateral NPT. Aggarwal et al. (74) reported that laparoscopy can be used to evaluate NPT, with not only an accuracy of more than 95% for testicular localization but also a higher success rate than traditional testicular fixation. However, some scholars hold different opinions. Igarashi et al. (75) conducted a retrospective study on the surgical methods applied in the treatment of 72 cases of NPT in 68 patients. A traditional inguinal incision and laparoscopic exploration were applied in the treatment of 28 and 44 testicles, respectively; the operation was successfully completed using both methods. The authors considered the incidence of high IAT to be relatively low and suggested starting with inguinal exploration for NPT; no extra-abdominal testis is found, and laparoscopic exploration can be performed. Bae et al. (76) retrospectively analyzed the cases of 183 consecutive patients with cryptorchidism from 2003 to 2012; there were 21 patients with unilateral and three with bilateral NPT. All patients successfully underwent surgery. The authors concluded that the traditional inguinal approach may be sufficient to treat unilateral NPT, whereas laparoscopy may be more suitable for patients with bilateral cryptorchidism.

High IAT remains a difficulty in the treatment of cryptorchidism because the blood vessels supplying the testes are short and testicular atrophy and other complications may easily occur after testicular descent in these cases. Fowler–Stephens orchiopexy (FSO), which was jointly proposed by Fowler and Stephens, is used to treat children with high IAT. In this technique, spermatic cord blood vessels that are too short to facilitate testicular descent into the scrotum are transected; i.e., the anatomical basis is that the internal spermatic cord artery, vas deferens artery, and levator testis muscle artery jointly provide blood to the testis (77). With the development of minimally invasive technology, Fowler–Stephens laparoscopic orchiopexy (FSLO) has gradually replaced traditional open surgery. Papparella et al. (78) retrospectively analyzed the data of 160 cases of laparoscopy for NPT. The results showed that among the 20 boys treated with FSLO, three boys had testicular atrophy, and the rest of the boys showed testicles in the scrotal position with a normal testicular texture. FSLO was used in 31% of IAT cases in the study, and the total success rate of this technique was 85%. Yu et al. (77) conducted a systematic review and meta-analysis of FSO in the treatment of high IAT. The results showed that the success rate of FSLO was significantly better than that of open FSO. Overall, the success rate of two-stage FSO was significantly higher than that of one-stage FSO, and the testicular atrophy rate after the treatment of high IAT with FSLO decreased year by year. Due to the limited data available, it is not possible to compare one- and two-stage open FSO. The authors concluded that two-stage FSLO is the first choice for the treatment of high IAT as it showed the highest success rate and the lowest atrophy rate.

**FIGURE 1:**
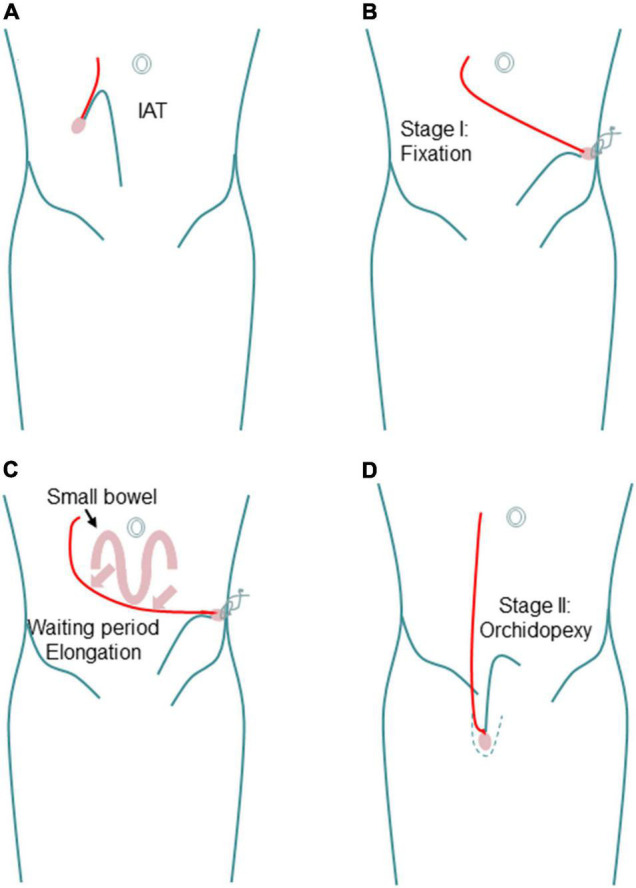
Schematic diagram showing the steps of Shehata technique. **(A)** Intra-abdominal testis. **(B)** Fixation in stage I. **(C)** Progressive gradual elongation during waiting period. **(D)** Scrotal position in stage II.

In 2008, Shehata first reported a new laparoscopic-assisted staged traction-orchiopexy for IAT (i.e., the Shehata technique), which gained scholarly attention (79) (Figure 1). In 2016, Shehata et al. (80) published the interim research results of this technique, reporting that the Shehata technique is a safe and successful method for the treatment of most cases of high IAT. However, testicles more than 4 cm away from the inner ring are not suitable for this technique. Our team first reported a comparative study of the FSLO and Shehata techniques in the treatment of high IAT in 2021 (4). The results show that the Shehata technique can effectively retain the main blood supply of the testis and vas deferens, with a high testicular survival rate and obvious advantages. The preliminary results show that the Shehata technique is safe, reliable, and effective in the treatment of high IAT in children. However, due to the short follow-up time, the long-term effects of the two surgical methods are not clear, and further research is needed in the future. For the treatment of high IAT, we recommend laparoscopic treatment. If the testis is within 4 cm from the inner ring, the Shehata technique can be applied; if this distance is greater than 4 cm, FSLO is recommended.

Another controversial issue is the need to remove testicular nubbin, the formation of which occurs under certain congenital conditions. In cases of nubbin, after the exploration for a clinically inaccessible testis, normal testicular tissue, including testicular remnants, nodules, or testicular/paratesticular tissue chains at the end of the spermatic cord, cannot be identified (1). The main reason for the debate about the treatment of nubbin is the variable incidence of living germ cells (GCs) reported in different studies, which has been reported to range between 0 and 16% (81). Only one case of intraductal germ cell tumor formation in testicular residue has been reported in the literature, and it was not supported by immunohistochemistry (82). However, Nataraja et al. (81) retrospectively analyzed the cases of 140 male children who underwent testicular residue resection, including 132 cases of inguinal testicular regression syndrome (TRS) residue resection and 8 cases of intraperitoneal residue resection. The results showed that one in 10 boys had GCs and that one in 4 boys had seminiferous tubules (SNTs). It was considered that the exploration and resection of inguinal testicular residues are necessary and might reduce the potential risk of malignant transformation in the future. However, Woodford et al. (83) studied 50 children with vanishing testes syndrome (VTS), which is defined by the presence of residual hypoplastic blood vessels entering the closed inguinal inner ring or distal gonadal tissue. Among them, 33 children were diagnosed with VTS under laparoscopy, and 31 testicles were examined by histopathology. The pathological results showed testicular nodules in 30 cases, with inactive testicular tissue, and no malignant tumors were found. Their research suggests that testicular nodules do not have viable GCs and do not need to be removed according to the malignant potential of residual testicular tissue.

In general, there are still many challenges in the treatment of high IAT. The various available surgical methods have their own advantages and disadvantages. It may be necessary to choose the appropriate surgical method according to the location and blood supply of the child’s testis.

#### Testicular transplantation

In 1976, Silber and Kelly first reported the application of microvascular anastomosis for high IAT in a child with prune belly syndrome, so that the external blood vessels could supply blood to the free testis (84). Tackett et al. (85) reported that the success rate of laparoscopic-assisted autologous testicular transplantation in the treatment of high IAT was 88%. Kelley et al. (86) reported the success of laparoscopic testicular free posterior microvascular anastomosis in 5 boys with high IAT between 23 months and 14 years of age. Perfusion scanning 5 weeks after the operation showed that the transplanted testis had sufficient blood circulation. Chao et al. (87) reported robot-assisted autologous testicular transplantation in an 18-year-old patient with left IAT. After more than 1 year of follow-up, the testis was still clearly visible in the scrotum and stable in size. Additionally, compared with the pre-operative measurement, there was no change in the serum testosterone level. The authors considered robot-assisted autologous testicular transplantation to be a feasible and effective method for the treatment of isolated IAT. With the development of microscopy, microvascular testicular autotransplantation is expected to become one of the better treatment methods for complex high IAT. In addition, experiments have shown that the use of stored spermatogonial stem cells for spermatogenic regeneration is successful in animal models. Sadri-Ardekani et al. (88) proposed that testicular biopsy can be used to extract testicular tissue from children for *in vitro* culture and storage. When children are infertile due to congenital developmental abnormalities such as cryptorchidism or testicular tumors, the stored testicular tissue can be used to mature spermatogenic stem cells and promote sperm formation for the purpose of fertility in the future. Fayomi et al. (89) confirmed that cryopreserved prepubertal testicular tissue can be autologously transplanted into the back skin or scrotal skin of castrated adolescent rhesus monkeys and can then mature to produce functional sperm. During the observation period of 8–12 months, the grafts grew and produced testosterone. Complete spermatogenesis was confirmed in all grafts at the time of recovery. Graft-derived sperm can fertilize rhesus monkey oocytes, leading to preimplantation embryo development, pregnancy, and the birth of healthy female infants.

#### Orchiopexy in neurologically impaired children

Barthord et al. (90) collected the electronic health record data of all male patients older than 7 years treated at a large multidisciplinary cerebral palsy clinic from 2000 to 2016 to determine the frequency of cryptorchidism and clinical comorbidities in the large cerebral palsy population. Finally, 839 cases were enrolled; in 553 cases, the testes were located in the scrotum, the testes were undescended in 185 cases, there were 38 cases of RT, and the status of the testicles was not documented in 63 cases. Their data show that the prevalence of cryptorchidism in men with cerebral palsy is approximately 10 times that in the general population but lower than the previously reported rate of 40–50% (91, 92). In cerebral palsy, cryptorchidism often occurs bilaterally, and this is related to the severity of the disease and the co-occurrence of other congenital abnormalities. In addition, other factors that may increase the risk of cryptorchidism in cerebral palsy include epididymal spasm, premature birth, and inguinal hernia repair in infancy. Harper et al. (93) conducted an e-mail survey on the surgical management of cryptorchidism in pediatric patients with severe encephalopathy at 27 French universities or general hospitals. The results showed that for palpable testis, two surgeons suggested hormone treatment as the first-line treatment, 11 doctors suggested orchidopexy in all cases, and 12 doctors suggested or at least discussed no treatment. For NPT, all surgeons recommended abdominal exploration and orchidopexy in most cases, but in the case of difficult orchidopexy and the presence of a contralateral testicle, five surgeons recommended orchiectomy. Springer et al. (94) conducted an online questionnaire survey of registered pediatricians in Austria and Germany to determine the pediatricians’ attitude toward the treatment of cryptorchidism (UDT) in boys with nerve injury (NIB). The results showed that 90.8% of doctors believed that UDT should be treated according to national guidelines, 8.6% of doctors believed that UDT should be treated according to the parents’ wishes, and only 0.6% of doctors preferred not to treat UDT. The study also found that tumor prevention, future sexual life, legal issues, anesthesia risk, and parental choice had a significant impact on the cognition of UDT. Finally, the researchers pointed out that from a pediatric perspective, UDT in NIB is an important issue and should be treated according to the guidelines. However, decision-making and selection of the best management strategy for UDT in NIB need further ethical discussion for optimization.

## Conclusion

**FIGURE 2:**
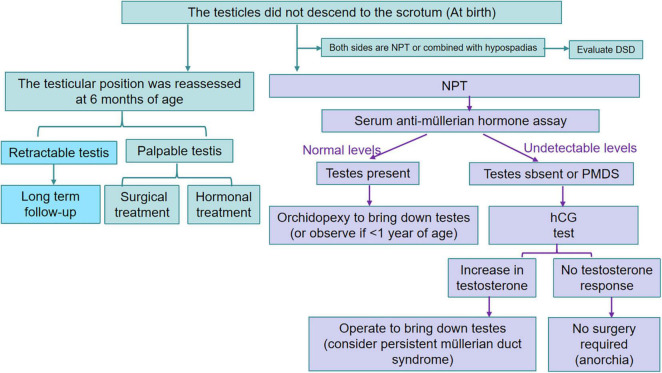
Flow chart of diagnosis and treatment of cryptorchidism.

In summary, cryptorchidism is a common congenital malformation in pediatric urology. At present, it is still controversial whether hormone therapy or surgery is the first choice for the treatment of cryptorchidism in children. It is recommended that professional pediatric surgeons screen and treat cryptorchidism children according to the established diagnosis and treatment process (Figure 2). At the same time, the awareness and education of children’s parents should be increased, the relevant knowledge level of personnel in primary healthcare institutions should be improved, and an optimized referral system should be established to effectively control delays in the surgical treatment of children with cryptorchidism. Patients with RT can develop into acquired cryptorchidism. The testicular position needs to be monitored until puberty is completed, but whether early surgical intervention is needed is not fully supported by the literature. Additionally, whether testicular nubbin requires routine resection remains controversial. With the development of microscopy, microvascular testicular autotransplantation is expected to become one of the better treatment methods for complex high IAT. In addition, with the developments in minimally invasive surgery, medical psychology, tissue engineering, and other disciplines, both the curative effect and quality of life can be improved for children with cryptorchidism.

## Author contributions

JL, GD, and NX designed the research. JL and WX performed the literature search and data analysis, drafted the work, and prepared the manuscript. XX, BS, and ZJ critically revised the work. NX and GD revised the manuscript. All authors contributed to the article and approved the final version of the manuscript.

## Abbreviations

NPT: non-palpable testis
RT: retractable testis
CHH: congenital hypogonadotropic hypogonadism
AMH: anti-Müllerian hormone
TDP: testicular distance to pubic bone
IGF-I: insulin-like growth factor-I
EDC: endocrine-disrupting chemical
TAI: testicular atrophy index
CT: computed tomography
MRI: magnetic resonance imaging
HPG: hypothalamic pituitary gonadal
hCG: chorionic gonadotropin
GnRH: gonadotropin releasing hormone
LHRH: luteinizing hormone-releasing hormone
WHO: Word Health Organization
GPR: growth percentage ratio
IAT: intra-abdominal testes
PPV: patent processus vaginalis
EAS: epididymal abnormalities
FSO: Fowler–Stephens orchiopexy
FSLO: Fowler–Stephens laparoscopic orchiopexy
TRS: testicular regression syndrome
GCS: germ cells
SNT: seminiferous tubules
VTS: vanishing testes syndrome.
